# Fragmentation and thresholds in hydrological flow‐based ecosystem services

**DOI:** 10.1002/eap.2046

**Published:** 2020-01-03

**Authors:** Amy Thomas, Dario Masante, Bethanna Jackson, Bernard Cosby, Bridget Emmett, Laurence Jones

**Affiliations:** ^1^ UK Centre for Ecology & Hydrology Environment Centre Wales Deiniol Road Bangor Gwynedd LL57 2UW United Kingdom; ^2^ Victoria University of Wellington Kelburn Wellington 6012 New Zealand

**Keywords:** ecosystem service, land use change, landscape configuration, nonlinear response

## Abstract

Loss and fragmentation of natural land cover due to expansion of agricultural areas is a global issue. These changes alter the configuration and composition of the landscape, particularly affecting those ecosystem services (benefits people receive from ecosystems) that depend on interactions between landscape components. Hydrological mitigation describes the bundle of ecosystem services provided by landscape features such as woodland that interrupt the flow of runoff to rivers. These services include sediment retention, nutrient retention and mitigation of overland water flow. The position of woodland in the landscape and the landscape topography are both important for hydrological mitigation. Therefore, it is crucial to consider landscape configuration and flow pathways in a spatially explicit manner when examining the impacts of fragmentation. Here we test the effects of landscape configuration using a large number (>7,000) of virtual landscape configurations. We created virtual landscapes of woodland patches within grassland, superimposed onto real topography and stream networks. Woodland patches were generated with user‐defined combinations of patch number and total woodland area, placed randomly in the landscape. The Ecosystem Service model used hydrological routing to map the “mitigated area” upslope of each woodland patch. We found that more fragmented woodland mitigated a greater proportion of the catchment. Larger woodland area also increased mitigation, however, this increase was nonlinear, with a threshold at 50% coverage, above which there was a decline in service provision. This nonlinearity suggests that the benefit of any additional woodland depends on two factors: the level of fragmentation and the existing area of woodland. Edge density (total edge of patches divided by area of catchment) was the best single metric in predicting mitigated area. Distance from woodland to stream was not a significant predictor of mitigation, suggesting that agri‐environment schemes planting riparian woodland should consider additional controls such as the amount of fragmentation in the landscape. These findings highlight the potential benefits of fragmentation to hydrological mitigation services. However, benefits for hydrological services must be balanced against any negative effects of fragmentation or habitat loss on biodiversity and other services.

## Introduction

Conversion of natural land cover to agriculture is a major driver of habitat and biodiversity loss globally, with associated loss of ecosystem services (ES; Foley et al. 2005). Such land cover change is likely to be accelerated in the foreseeable future as a result of increased conversion of natural land to agricultural production in order to satisfy global food demand (Tilman et al. 2011). The degree of change and the spatial pattern of this change may be important for ES as well as biodiversity.

Spatial pattern of features in the landscape has a considerable influence on biodiversity and ES provision, and is particularly important for ES involving flow or movement between landscape components (Mitchell et al. 2015a, Verhagen et al. 2016, Lavorel et al. 2017). Landscape configuration is defined as the spatial arrangement of landscape components, and includes a range of concepts including fragmentation, spatial arrangement and connectivity. Fahrig (2003) note that fragmentation (patch size, edge effects, and core area) is an important determinant of impacts on biodiversity. The spatial arrangement and connectivity of patches within the wider landscape are also ecologically important, and will vary according to the pattern of fragmentation (Didham 2010). These multiple components of landscape configuration can affect ES in a number of ways.

Fragmentation also tends to reduce connectivity between habitat patches, with negative impacts on biodiversity (e.g., Keller and Largiader 2003, Tabarelli et al. 2008). This decreased connectivity between patches is usually assumed to have a negative effect on ES provision, primarily for mobile agent‐based services (MABES; Kremen et al. 2007). Mitchell et al. (2013) concluded that changes in connectivity between patches may have different effects on other types of services with different dependencies on habitat matrix interactions and exchanges. However, there are examples where fragmentation can be beneficial. Robinson et al. (2009) found increased carbon storage services per ha with forest fragmentation due to edge effects increasing tree growth. For hydrological services, spatial arrangement and proximity to preferential flow pathways (rivers) may be particularly important; for example, it has been shown that the distance of woodland from a stream can have a greater impact on sediment loss from a catchment than physical controls on soil erosion (Chaplin‐Kramer et al. 2016).

Landscape features that interrupt the overland transport of water and diffuse pollution to the watercourse provide a hydrological mitigation service (Jackson et al. 2008). They do this by increasing infiltration of water and soluble pollutants into the soil, and by trapping sediment. Riparian corridors of long grass, forest or other seminatural vegetation can reduce sediment and soluble pollutant losses in surface runoff by 50–80% (van Dijk et al. 1996, White and Arnold 2009, Zhang et al. 2010). Verhagen et al. (2016) found that modeling landscape configuration was necessary for accurate representation of hydrological services, however, this has been little studied in the context of ES (Mitchell et al. 2013). In the hydrology literature, modeling of overland flow has shown that fragmentation has a positive relationship with infiltration (Ziegler et al. 2007). However, Ziegler et al. (2007) did not represent flow accumulation and were therefore not able to account for the spatial arrangement of patches relative to upslope area. In order to better quantify the mitigation service, it is necessary to calculate the area of runoff accumulation upslope of features that provide a mitigating service. Taking this into account, in addition to spatial arrangement and connectivity of patches, allows us to better understand how landscape configuration affects hydrological mitigation.

Despite this evidence, it is surprisingly common for ES mapping to assume constant values for ES provision according to land cover, and take no account of their position in the landscape (Lautenbach et al. 2019). Since a major application of ES assessment is to provide guidance to policy makers, this may result in planned land cover changes that miss opportunities for ES gains, or worse, create unexpected detrimental impacts. Better understanding and representation of the influence of landscape configuration is therefore critical to mapping ES for policy and landscape planning, as well as understanding the impacts of future land cover change.

There are a number of approaches to explore these issues. For example, comparison across multiple real catchments, as applied in Verhagen et al. (2016), is useful in highlighting the importance of landscape configuration, but cannot isolate the impacts of landscape configuration on ES, since variation in land cover metrics is confounded by variation in topography. Configuration has also been investigated using virtual landscapes: using checkerboard patterns, e.g., Mitchell et al. (2015b), which do not consider preferential flow pathways; or using designed individual virtual landscapes (Adriaensen et al. 2003, Robinson et al. 2009) that enable greater complexity to be considered but limit sample size and associated statistical analysis. Neither of these approaches allows statistical assessment of the impact of landscape configuration on mitigation services independent of topography. There remains considerable scope for further refining approaches for the creation of virtual landscapes and applying these with models that can represent accumulation of flow and diffuse pollutants.

In this study, we aimed to explore the importance of landscape configuration as a control on ES provision using mitigated area as a proxy for a bundle of services that are strongly affected by abiotic hydrological flow pathways: potential mitigation of runoff, sediments, and diffuse pollution). In this context, we consider a bundle to mean those ES that covary spatially due to shared processes and controls underpinning them, as applied in Mouchet et al. (2017), rather than bundles with observed correlations in value, or bundles of similar type, e.g., provisioning, regulating. By modeling in virtual landscapes based on real topography we were able to explore the interaction between total area of natural landcover (in this case woodland) and the number of patches and edge density. By modeling many combinations of area and patch number, we can also separate these effects from variation in other parameters such as topography and the resulting natural variation in flow pathways. In this paper, we explored the following hypotheses:

1. Intuitively, we expect an increase in the area providing a mitigating service to increase the overall provision of service. For an individual patch, spatial location relative to surface water flow accumulation will determine the service provision. Across all test landscapes, we expect that a greater number of patches will lead to greater likelihood of some being well located for ES provision. Therefore, we also hypothesize a correlation between patch number and mitigation ES.
2. These relationships may be nonlinear or subject to thresholds since, as the area providing mitigation increases, new provision is more likely to overlap with existing provision.
3. Due to the importance of flow accumulation and proximity to flow pathways, we expect topography and stream networks to be important controls on mitigation. We would expect shorter mean distance to stream to correlate with higher mitigation, since upslope area is likely to be greater. We also expect influence from topography and stream networks to lead to variation in relationships between catchments with different characteristics.

## Methods

### Study area(s)

**Figure 1:**
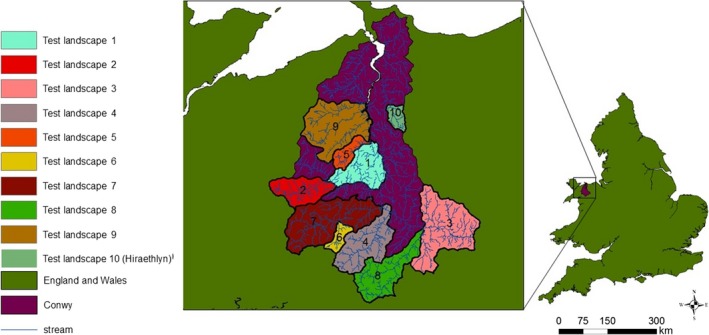
The Conwy catchment, North Wales, UK, showing location of the study sub‐catchments.

The Conwy catchment in North Wales is 580 km^2^ in area, of which 380 km^2^ is above the tidal limit of the River Conwy. Elevation ranges from 0 to 1,064 m above sea level, with large areas of strongly or steeply sloping land. Dramatic topography generates orographic rainfall, and annual precipitation varies from 500 mm at sea level to 3,500 mm at higher altitudes. The majority of the land cover is grassland (66% of which 25% is improved grassland, i.e., fertilized and drained), followed by heather moorland and upland acid grassland. There is around 5% broadleaved woodland and 10% coniferous woodland (Morton et al. 2007). More information on the Conwy catchment can be found in Emmett et al. (2016). For this study, we selected 10 sub‐catchments, designed to represent gradients in stream hierarchy, size, and topography. We initially focus on the 8‐km^2^ sub‐catchment highlighted in Fig. 1, as “Test landscape 10 (Hiraethlyn)”. This sub‐catchment is broadly representative of the Conwy as a whole, with both steep and gentle slopes, numerous peaks, and elevation ranging from 60 to 380 m above sea level. Later in the study, we expanded our analysis to 10 sub‐landscapes.

### Simulating virtual landscapes with landscapeR

In order to measure the effect of landscape composition on ES provision, a computer simulation was set up to vary land cover under controlled conditions, but superimposed on real topography. To do so, we developed a new tool for R (R Core Team 2013) called landscapeR, available as an R package (Masante 2016). Unlike existing tools (e.g., G‐RaFFe; SimMAP; Qrule; and others described in Pe'er et al. 2013), landscapeR is able to work on real landscapes, provided as categorical maps in input, and to simulate changes in their land cover, based on spatial directives only. For this simulation, in order to create relatively simple landscapes consisting of varying landscape configurations of two components, we artificially set the whole area as improved grassland and then superimposed patches of woodland within this landscape, in controlled combinations of patch area and number of patches. In landscapeR, the basic function to create patches implements an agent‐based‐modeling algorithm, which expands the patch starting from a seed point in the landscape. This approach allows for a higher control over patch features, compared to available stochastic simulation tools (such as SimMAP). In order to limit the number of potential explanatory variables, we reduced impact of patch shape by avoiding creation of linear patches; instead, patches were set to grow in roughly uniform circles around the random seed points. However, as patches start accumulating in the landscape, they merge and so the complexity of patch shapes inevitably increases. Simulated landscapes were established to answer two questions: firstly to identify the relative controls of patch area and fragmentation on service provision, and secondly to identify catchment feature controls on these relationships.

In the first stage of the simulation, we created 100 simulated landscape configurations of woodland patches and improved grassland for the Hiraethlyn sub‐catchment, allocating patches randomly up to the user input number of patches and area of coverage. Woodland patches were set up with the following rules: landscape proportion 10%, 25%, 50%, 75% and number of woodland patches 1, 2, 4, 8, 16. By creating landscapes with each combination of patch number and landscape proportion, we were able to separate impacts of fragmentation per se and forest area, as advocated by (Fahrig 2003).

For each combination of landscape proportion and number of patches, the forest patches were placed randomly within the landscape, to create five replicates. Since patch placement was random, some combinations specified as the seed were not achieved in the output, due to merging of patches, reducing the actual number of patches. To minimize merging, when allocation resulted in a lesser number of patches than seeded, the process was repeated until the seed number was reached or the highest in 100 trials. All simulations achieved the desired landscape proportion, which was given preference over achieving the number of patches. Merging may therefore result in a negative correlation between landscape proportion and number of patches across the data set, and we must take this into account when analyzing the outputs.

In the second phase of analysis using all 10 sub‐landscapes, the landscape generation approach was repeated for all 10 test areas. For this phase, landscape proportion and patch number were not pre‐set, so the landscapes generated have random numbers of patches and fall along a continuum from 0% to 100% woodland, instead of being clustered around set landscape proportions. Over 700 landscape configurations were generated for each of the 10 test landscapes.

### LUCI ES model and runoff mitigation

The Land Utilization Capability Indicator (LUCI) model is an integrated land management decision support model that is increasingly being applied to map areas providing ecosystem goods and services (Sharps et al. 2017). The LUCI model simulates a range of ES and trade‐offs between these, however, in this study we focus on runoff mitigation, retention of sediments, and diffuse pollution as these are often provided by the same landscape features and respond in the same way to spatial context. This “hydrological mitigation” bundle of services is dependent on the movement of water and the sediment and solutes it carries over the landscape, and is therefore sensitive to landscape configuration.

To model hydrological mitigation ES, this spatial modeling tool applies distributed topographic routing to simulate flow accumulation over the landscape, and track movement of water, sediment, and diffuse pollutants to streams (Jackson et al. 2013, Trodahl et al. 2017). The tool identifies features such as woodlands or wetlands that enhance retention and infiltration of water and diffuse pollution, and increase available soil water storage capacity (as described in Jackson et al. 2008, Marshall et al. 2009). The model is based on evidence that the reduction of rapid overland flow by these features can deliver runoff mitigation ES through retention of sediments and diffuse pollution (Jackson et al. 2008). There may be further benefits for flood peak mitigation (Jackson et al. 2008), though these may only be attained at small or sub‐catchment scales (Dadson et al. 2017).

The tool is able to quantify the mitigated area for individual features, using an approach primarily based on flow accumulation mapping (Jackson et al. 2008). Mitigation is assumed to take place when flow occurs from non‐mitigating land cover into mitigating land cover, as per Ziegler et al. (2007). The area from which water is routed through mitigating features (i.e., the upslope area) is identified and mapped as receiving mitigation; here, we use this “mitigated area” as a proxy measure for the provision of mitigation ES. Thus, this aspect of service provision of an individual patch is controlled by location in the landscape relative to modeled accumulation of overland flow and near‐surface soil flow. By investigating mitigated area at the landscape scale across a large number of landscape configurations for the same topography, we can perform statistical analysis to generalize the effects of fragmentation and forest area on provision of mitigation services.

Mitigated area provides a useful proxy measure: the amount of retention of diffuse pollution or entrained sediment will be dependent on loading for a given runoff event, as well as site factors such as slope and soil type (e.g., Weller et al., 1998), which cannot be represented with a generalized ES model. Any flood peak mitigation effects will also be dependent on synchronicity of hydrograph peaks between tributaries (Saghafian and Khosroshahi 2005), and the scale at which flood risk is a concern (Dadson et al. 2017). Nonetheless, the LUCI model has shown good performance in validation against observed data for simulated flow and N concentration in streams at the national scale in Wales (see supplementary material in Sharps et al. 2017). Performance will be dependent on accuracy of input data on agricultural pollutants and topography (a 5‐m DTM [NextPerspectives 2014] is available for the UK, which is hydrologically corrected as part of model processing).

### Analysis of model outputs

The creation of detailed high‐resolution landscapes, combined with model functionality to loop through multiple scenarios, enables generation of a large sample size of scenarios for statistical analysis. Statistical analysis of the output was a two‐stage process; stage 1 explored controls for a single catchment, while stage 2 investigated which catchment characteristics governed those relationships.

### Stage 1: Identifying influence of forest area, fragmentation, and landscape configuration on service provision and thresholds in these relationships

**Figure 2:**
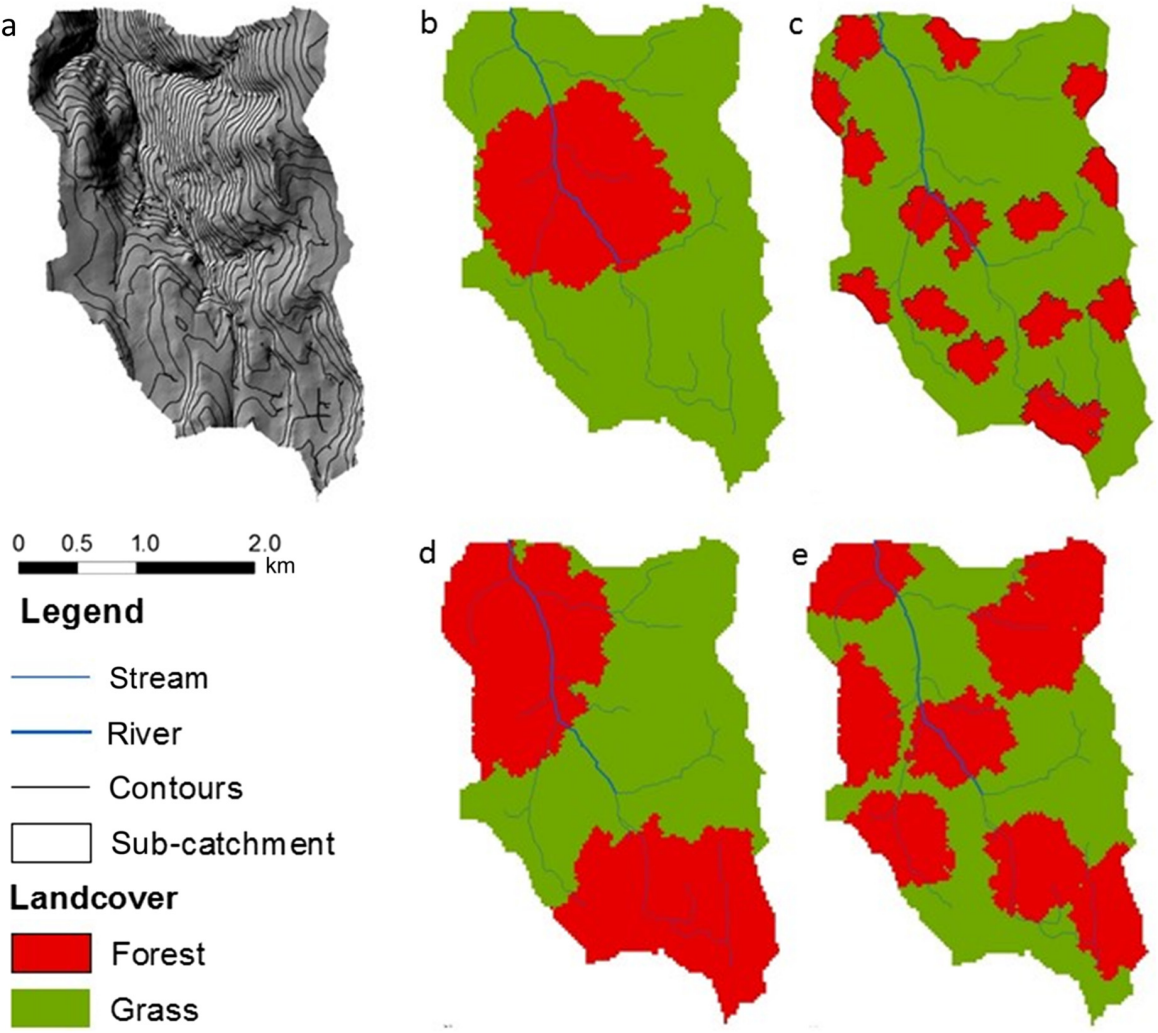
Selected examples of the random landscape compositions generated for the Hiraethlyn catchment: (a) topography (b) forest covering 25% of the catchment in one patch, (c) forest covering 25% of the catchment in 15 patches, (d) forest covering 50% of the catchment in two patches, (e) forest covering 50% of the catchment in six patches.

We analysed the data set of landscape statistics and model output to assess the relationship of hydrological mitigation ES (assessed by mitigated area) with patch size, total forest area, and metrics of landscape composition. The metrics tested are explained in Table 1 (Fig. 2). Landscape metrics were computed through SDMTools (VanDerWal et al. 2014). We used the data set to construct and compare regression models, to identify the best landscape metrics for predicting total area receiving mitigation. These metrics were tested individually and combined, and the regression models were compared based on AIC and the significance of individual terms, since variation in degrees of freedom negates the approach of an *F* test. Correlation between variables is assessed in Appendix S1: Table S1 and, where necessary, models were developed to account for correlation between key controlling variables (e.g., landscape proportion and patch number) to ensure that the impacts could be separated. This analysis enabled exploration of controls on mitigation, and identification of the best single term for predictions. The use of seed constraints enabled us to assess the importance of landscape proportion and patch number as factor variables.

**Table 1:** Explanation of metrics tested in regression models for the Hiraethlyn sub‐catchment

Metric tested	Explanation
Mitigated area: the area upslope of woodland patches once hydrological routing has been accounted for This is the proxy metric to indicate provision of mitigation ecosystem services (ES), and is the dependent variable in each analysis here.	Indicates the area from which runoff of pollutants and sediment accumulates before overland flow is interrupted by a forest patch. Due to infiltration and retention by the forest patch, these pollutants may not reach the watercourse, hence a mitigation ES is provided
Patch density: number of patches divided by the total landscape area to give a value that can be compared between test landscapes.	Indicates the level of fragmentation of the habitat of interest. This tests the effects of fragmentation without accounting for habitat loss; e.g., Fig. 2c has much greater patch density than Fig. 2b even though both have the same area of forest.
Landscape proportion: area of natural land cover (in this case woodland) divided by the total landscape area to give a value that can be compared between test landscapes.	Indicates the relative area of the land cover of interest. This tests the effects of woodland loss without accounting for fragmentation; e.g., Fig. 2d and e have much greater LP (50%) than 2b and 2c (25%).
Total edge: total length of boundary between land cover of interest and other land cover types. This may be thought of as the perimeter of all patches. Edge density: total edge divided by landscape area. This provides a measure of fragmentation that can be compared between landscapes.	These are measures of fragmentation that are indicative of a specific control on service provision: for hydrological services modeled here, the transition between land cover types is where the service provision can occur. These edge metrics correlate strongly; for comparability across landscapes only edge density was used.
Mean distance to stream: overland flow distance from the patch to the stream, calculated as an average across all pixels in a habitat patch.	A measure of patch distribution relative to preferential pathways; this has been shown to be an important metric for creation of sediment runoff in other contexts (Chaplin‐Kramer et al. 2016).
Euclidean nearest neighbor distance: the shortest straight‐line distance between the focal patch and its nearest neighbor of the same class.	Euclidean nearest neighbor provides a measure of the even‐ness of the spatial distribution of patches, to assess the relevance of this compositional metric.
Coefficient of variation of Euclidean nearest neighbor distance: used here to evaluate at landscape scale, a small value implies relatively even dispersal (low standard deviation relative to mean).	Coefficient of variation of Euclidean nearest neighbor distance is the most useful metric to indicate evenness at landscape scale.

### Stage 2: identifying catchment controls on the relationships between forest area and fragmentation on service provision

Having identified the best single metric, we then performed statistical analysis to understand why the relationship might vary between catchments, looking for explanatory variables for variation in both the coefficient and the scatter in the observed relationship. We tested possible predictors from the range of measurable landscape characteristics. The coefficient and mean square error of the relationship to the metric identified in stage 1 were extracted for each test landscape. Regression analysis used (1) the coefficient and (2) mean square error as the response variable for two separate regression equations. Data were not normally distributed, so these regression models were fit with a tweedie distribution. The landscape characteristics assessed as the predictor variable(s) included total area, drainage density, total stream length, standard deviation of slope, mean stream order, sum stream order, and standard deviation of aspect. These variables were intended to capture information on the range of catchment size and topographic complexity. Stream order is indicative of the hierarchy of tributaries in the catchment; a smaller mean suggests a greater proportion of minor tributaries, and may indicate a more complex topography, while sum of stream order may correlate with catchment size, with added influence from drainage density and catchment complexity. Values were calculated using the Strahler approach (Strahler 1957).

## Results

### Stage 1: Identifying influence of forest area, fragmentation and landscape configuration on service provision, and thresholds in these relationships

**Figure 3:**
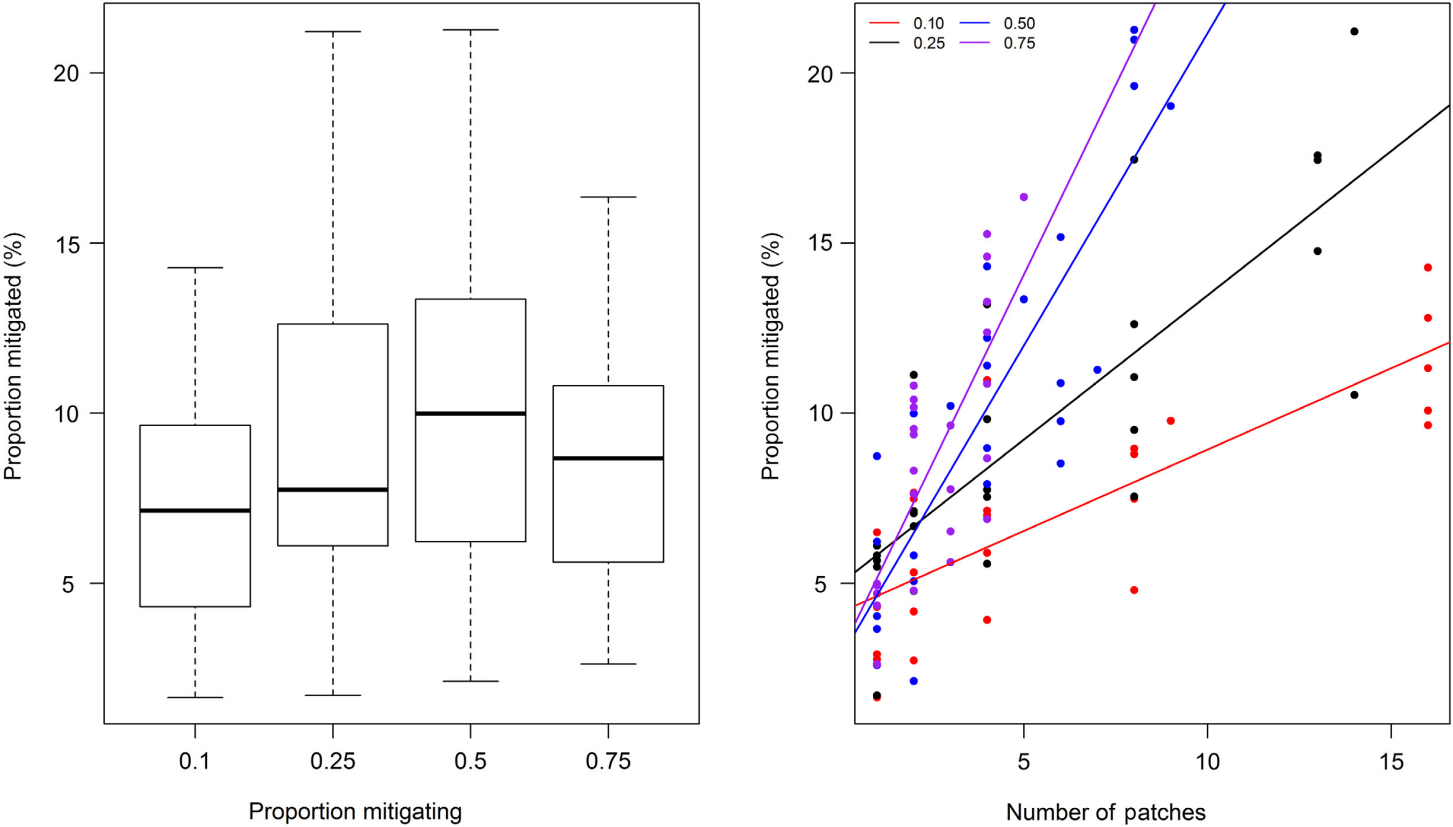
Plots for simulations of the Hiraethlyn sub‐catchment showing how mitigated area (the proxy metric to indicate provision of mitigation ES, expressed as percentage of catchment receiving mitigation) varies with (a) landscape proportion providing mitigation (i.e., occupied by woodland), and (b) number of woodland patches providing mitigation. Color denotes the landscape proportion occupied by woodland, which can be seen to alter the relationship.

Regression models show that landscape proportion was not a good predictor of mitigation when assuming a linear relationship (Table 2, Model 1). This may reflect nonlinearity and thresholds in the relationship, as hypothesized. Fig. 3a shows that there may be some positive trend up to a landscape proportion of 50% forest area for the Hiraethlyn (see also Appendix S1: Figs. S1, S2). With further increase in landscape proportion, the proportion of grassland receiving mitigation starts to decrease, despite the median area of grassland mitigated being relatively small at around 10%.

**Table 2:** Regression models for the Hiraethlyn catchment relating service provision to landscape composition

Models of area mitigated	Estimated coefficient	Deviance explained (%)	*r* ^2^	AIC	Significance
1. Landscape proportion	0.1685	1.27	0.00259	85.8	0.265
2. Landscape proportion (with smoother)	+	15.9	0.114	77.8	0.0133*
3. Patch density	17700	35.8	0.351	42.7	<0.001***
4. Edge density	0.0001340	57.6	0.572	1.25	<0.001***
5. Mean distance to stream	0.000475	0.465	−0.00551	86.6	0.5
6. Coefficient of variation of Euclidean nearest neighbor distance	−0.00251	2.47	0.0147	84.5	0.119
Additive models					
7. Patch density	21400	47.6	0.465	24.4	<0.001***
Landscape proportion	0.537				<0.001***
8. Edge density	55.48	67.6	0.67	−23.7	<0.001***
Landscape proportion	−0.550				<0.001***
9. Edge density	56.3	70	0.694	−31.3	<0.001***
Mean distance to stream	−0.00281				<0.001***
10. coefficient of variation of Euclidean nearest neighbor distance (CV.ENN)	−0.00001	57.6	0.567	3.251	0.993
Edge density	44.5				<0.001***
11. Edge density	36.01	65.6	0.649	−17.8	<0.001***
Patch density	9434				<0.001***
With interaction term					
12. Patch density	3130	67.9	0.669	−22.5	0.296
Landscape proportion	−0.288				0.0413*
Interaction term	85200				<0.001***
13. Edge density	65.45	68.3	0.673	−23.8	<0.001***
Landscape proportion	−0.247				0.297
Interaction term	−24.4				0.159
14. Edge density	70.4	71.3	0.704	−34.0	<0.001***
Mean distance to stream	−0.000821				0.429
Interaction term	−0.161				0.0359*
15. Coefficient of variation of Euclidean nearest neighbor distance (CV.ENN)	−0.00413	59.1	0.579	1.51	0.0888
Edge density	33.1				<0.001***
Interaction term	0.385				0.0587
16. Edge density	24.5	67.4	0.664	−21.01	<0.001***
Patch density	−2757				0.63
Interaction term	857,100				0.02*
17. Number of patches smoothed by landscape proportion (as factor)	+	70	0.663	−13.27	<0.001***

*Notes*

The best single metric (edge density) was combined with other metrics to see if they became important in a combined model.

Units are as follows: patch density, number of patches/total area in 5 × 5 m pixels; landscape proportion, m^2^/m^2^; edge density, m/m^2^; mean distance to stream, m; coefficient of variation of Euclidean nearest neighbor distance, %.

*P ≤ 0.05; ***P ≤ 0.001.

Hence we tested for a nonlinear relationship and found that landscape proportion is a significant control on mitigation area once the relationship is allowed to follow a curve (Table 2, Model 2). Further investigation across all 10 test catchments suggests a consistent relationship that follows a hump‐shaped curve, with the inflection point varying according to catchment size (Appendix S1: Figs. S1, S2); broadly speaking, larger catchments had peak service provision at a greater landscape proportion and the proportional service provision achieved was lower.

Our findings indicate that greater fragmentation tends to increase the mitigated area (Fig. 3b, Table 2, Model 3 and 17). Comparing trends between levels of landscape proportion (indicated by color in Fig. 3b, Model 17), statistically significant positive correlations were observed for each, and the trend is steeper at higher landscape proportion. From Table 2, we see that both patch density and landscape proportion were important in a combined additive model and, if an interaction term is included, only the interaction term has strong significance (Model 12, hence the trend lines cross in Fig. 3b). The impacts of this interaction may be significant in terms of area required to deliver service; e.g., in the Hiraethlyn test catchment, it was possible to achieve a mitigated area of around 15% by having 50% of the area as woodland when distributed across seven patches, but the same amount of mitigated area could be achieved with only 25% of woodland distributed across 12 patches. Similarly, it was possible to achieve the same level of mitigation with less fragmentation at higher landscape proportion (indeed higher levels of fragmentation could not be achieved, due to overlapping of patches, as reflected in the correlation between landscape proportion and patch density [Appendix S1: Table S2]).

**Figure 4:**
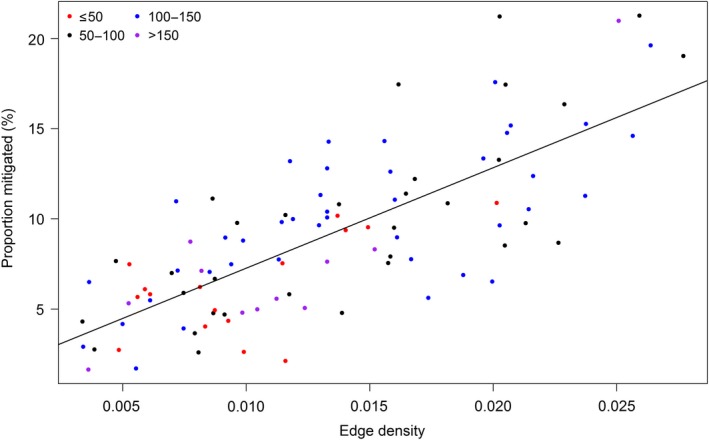
Plot for the Hiraethlyn sub‐catchment showing edge density of woodland patches (sum of patch boundaries divided by landscape area) providing mitigation, against mitigated area (the proxy metric to indicate provision of mitigation ecosystem services (ES), expressed as percentage of catchment receiving mitigation). (a) Color denotes the landscape proportion occupied by woodland, which has some effect on the relationship, but is not a statistically significant factor. (b) Color denotes the mean distance of patches from nearest stream, which was statistically significant when included in an additive model.

Table 2, Model 4 indicates that edge density was the best single metric to explain variance in our data set. Edge density provides a measure of fragmentation in terms of patch boundary (edge length) divided by landscape area, which can easily be compared between landscapes. Increasing the edge density of the woodland patches providing mitigation tends to increase the area of grassland receiving mitigation (as shown in positive coefficient in Table 2, Model 4, and the trend line in Fig. 4). Therefore edge density is a useful single metric to predict mitigation ES. Edge density also has theoretical importance as a control on hydrological service provision; since mitigation occurs where flow occurs from non‐woodland into woodland. A combined model (Table 2, Model 13) suggests that landscape proportion does not affect the gradient of the relationship, although landscape proportion was significant in the additive model (Model 8). Visualization in Fig. 4 suggests that, as area providing mitigation increases, the intercept decreases, which may reflect the trend of decreasing area available to receive the service. Positive correlation between edge density and landscape proportion complicates the process of interpreting this trend.

Other landscape metrics tested were mean distance from stream and coefficient of variation of Euclidean nearest neighbor distance, which are often considered strong controls. These only showed a relationship with service provision when combined with edge density (Table 2, Models 9, 10, 14, and 15). Combination of these metrics with patch density and landscape proportion (not shown) gave poor performance, and the additional landscape metrics were not significant in the models.

When combined models were tested, edge density and mean distance to stream gave best performance when compared on variance explained (70%) and AIC. This suggests that distance to stream has some importance in predicting the remaining variance in the data once edge density has been accounted for. Exploration of the data in Fig. 4b indicates that landscapes with lowest average patch distance from stream (≤50 m) tended to have low edge density, hence this confounding effect may mask the influence of distance to stream in the single variable model.

The coefficient of variation in nearest neighbor distance was not statistically significant in any combined model; this reflects the greater importance of preferential flow pathways than patch dispersal metrics for hydrological ES.

### Stage 2: Identifying catchment controls on the relationships between forest area and fragmentation on service provision

**Figure 5:**
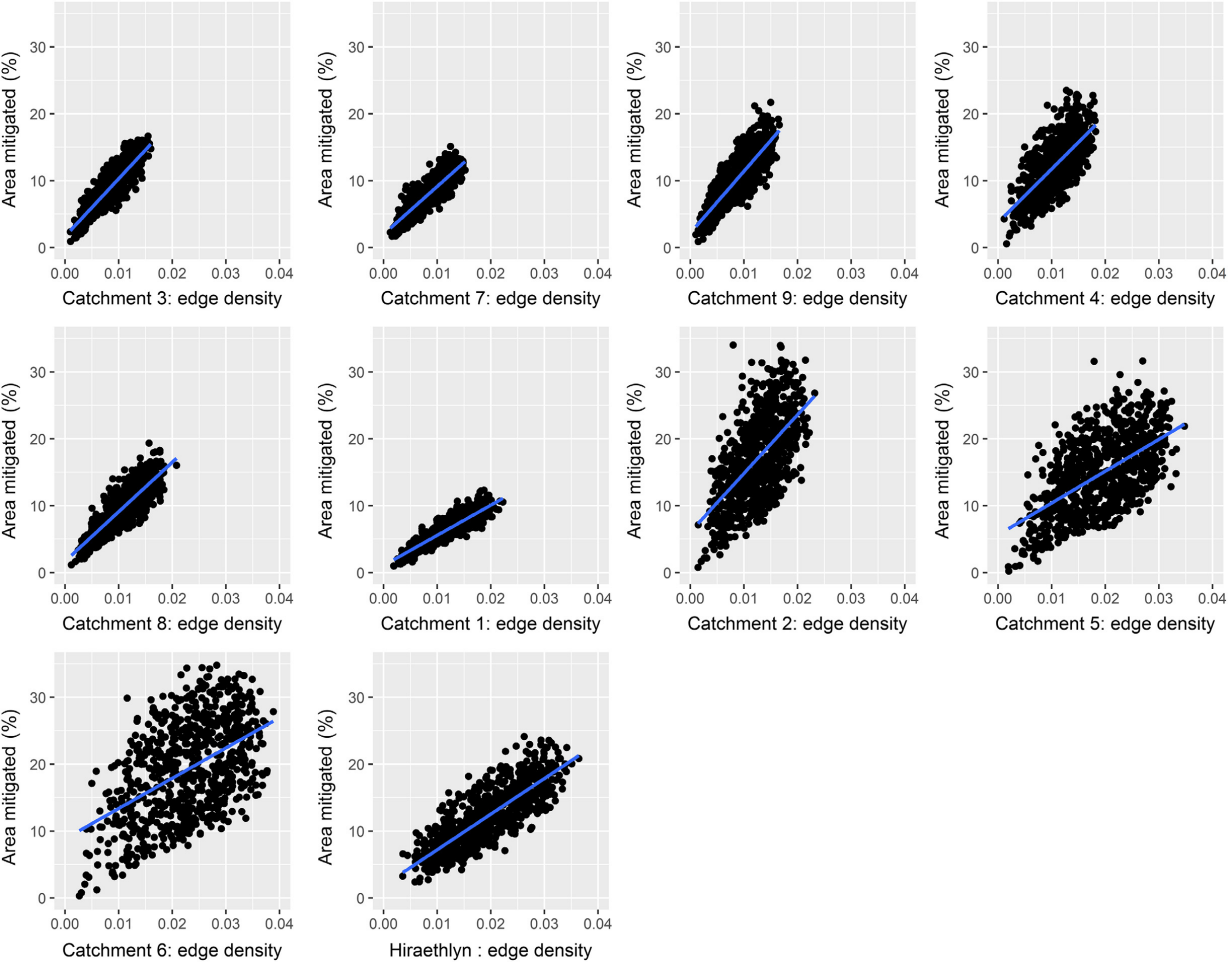
Plot showing edge density of patches providing mitigation (sum of patch boundaries divided by landscape area) against percentage of catchment receiving mitigation for each of the 10 study test landscapes. Landscapes are arranged in order of decreasing size.

When tested across multiple catchments, the positive relationship between increasing edge density and mitigated area holds true (Fig. 5, Appendix S1: Table S4). However, there is variation among catchments in the amount of scatter as well as in the slope of the relationship. This variation is likely driven by differences in catchment characteristics. In order to explore the variation in the relationship, we constructed models for slope and scatter using landscape descriptive variables in Table 3.

**Table 3:** Analysis of factors governing slope and scatter in the relationship between edge density (the best predictor in Table 2) and area mitigated (as %) across the 10 test landscapes indicated in Fig. 5

Edge density model	Estimated coefficient	Deviance explained (%)	*r* ^2^	Significance
Models of coefficient of mitigation				
Sub‐catchment area	9.47 × 10^−9^	51.6	0.433	0.017*
Drainage density (km/km^2^)	−0.619	12.2	0.0258	0.316
Slope SD	−0.0385	9.93	−0.00585	0.370
Slope mean	−0.0251	10.5	−0.00559	0.348
Stream order mean	0.226	1.31	−0.122	0.708
Stream order sum	1.23 × 10^−5^	31	0.174	0.080
Aspect SD	0.0127	10.4	−0.0593	0.310
Models of MSE of mitigation				
Sub‐catchment area	−4.27 × 10^−8^	53.5	0.373	0.008**
Drainage density (km/km^2^)	4.17	29.9	0.220	0.083
Slope SD	0.298	25.1	0.0534	0.072
Slope mean	0.224	53.5	0.652	0.016
Stream order mean	−6.14	60.9	0.513	0.003**
Stream order sum	−7.62E‐05	61.9	0.388	0.003**
Aspect SD	0.0124	1.44	−0.0890	0.799

*P ≤ 0.05; **P ≤ 0.01

The slope of the relationship is correlated with catchment size. Larger catchments show a greater increase in mitigated area per unit increase in edge density, as shown by the statistically significant positive model coefficient in Table 3. Drainage density (indicative of catchment complexity) was not a statistically significant predictor of the coefficient of relationship between ED and mitigated area. Other factors such as mean slope angle, variation in slope angle, and stream‐order metrics were also not significant.

The scatter in the relationship appears to be related to both catchment size and complexity. Larger catchments with more streams should see slightly less scatter in the relationship between ED and mitigated area, since Table 3 shows that sub‐catchment area and measures of stream order were all significant predictors of the mean square error in the relationship. Although drainage density was not significant, there was, however, a significant negative coefficient for mean stream order, suggesting that catchments with more complex topography and a stream hierarchy weighted towards small streams (implying greater overall complexity) will have more scatter in the relationship. Complex topography may be expected to increase scatter due to the greater importance of patch placement, which differs randomly between test landscapes.

## Discussion

This work shows a significant influence of landscape configuration on a bundle of ES that are affected by hydrological flow pathways. By using a virtual landscape approach to isolate the impact of landscape configuration from other controls such as topography, and by analyzing a large number of landscape configurations, we were able to systematically assess the effects of fragmentation and test the factors that explain the observed trends. Results suggest that both increasing the area of forest providing a service (up to a threshold) and increasing the fragmentation of that forest will increase service provision. Characteristics such as catchment size and stream length may be important controls on these relationships.

### Influence of forest area, fragmentation, and landscape configuration on service provision

Our findings that both increased area (below 50%) and increased fragmentation lead to greater service provision support our primary hypothesis. Both area of forest and patch number tend to increase service provision and the interaction of these two factors is synergistic. Thus we generally see an increase in mitigated area with increase in fragmentation, but forest fragmentation accompanied by forest loss might be expected to reduce service provision.

Using hydrological modeling of overland flow, it was shown by Ziegler et al. (2007) that fragmentation increases mitigation (for locally generated runoff only), due to greater frequency of transition from source areas to sink areas. Here we extend that conclusion to suggest that, once landscape configuration has been accounted for in terms of flow accumulation and once abiotic flow pathways have been accounted for, fragmentation is still beneficial to this service of hydrological mitigation. However, it is important to note that fragmentation is often accompanied by reduction in area of natural landcover. Therefore, benefits for these ES must be balanced against any negative effects of fragmentation or habitat loss on biodiversity and other services.

Fragmentation is inherently related to landcover diversity, and the edge contrast between landcover types is important for a range of ES. Ecosystem service bundles with differing controls may be expected to respond differently. For example, negative impacts of fragmentation have been recorded for mobile‐agent‐based ES (as reviewed in Mitchell et al. 2013), and for generalized distance‐dependent services with no directionality and a requirement for a certain patch size to sustain the service (e.g., Mitchell et al. 2015b). Both these services are more affected by distance than by preferential pathways.

### Existence of thresholds in the relationships of forest area and fragmentation with service provision

Provision of ES can be expected to be nonlinear and subject to thresholds (Costanza et al. 1997, Koch et al. 2009). For example in our simulations, the maximum mitigated area was obtained under intermediate levels of woodland in the landscape, hence care should be taken not to assume that increasing landscape proportion (area of woodland in this case) will automatically increase service provision. This is particularly true where the definition of the service requires an adjacent area or population to receive it. In this study the benefit to be gained by planting additional woodland will depend on the starting point on the curve, i.e., how much woodland is already there. Beyond that threshold, random placement of new woodland is more likely to occur on land that is already mitigated by woodland elsewhere, and thus may lead to a reduction in mitigated area. Conceptually, mitigating area may also be thought of as mitigated, i.e., the area under woodland is itself mitigated, so if a combined metric of woodland providing mitigation and mitigated area was used, this threshold would be indicated by a levelling out of benefit, rather than a reduction. However, in the context of woodland providing mitigation for another type of land use, it is helpful to consider the threshold as the point beyond which further increase in randomly placed afforestation generates diminishing returns. This analysis makes the assumption that each patch is 100% efficient at mitigation. In reality, not all woodland patches provide complete mitigation (Ziegler et al. 2006), therefore new woodland creation within an area that is already mitigated may further increase the quality of mitigation service under some conditions.

Mitchell et al. (2015b) found that with greater fragmentation, peak ES provision occurred at a higher landscape proportion, around 70%. This relates to their assumption that, for the services assessed in their study, provision is correlated to fragment size, and that small fragments lose the capability to provide ES. It has been suggested that, for some ES, provision may be reduced or lost below certain patch size thresholds, due to detrimental effects on underpinning ecology (Groffman et al. 2006). Kremen et al. (2007) note that for pollinator services, size thresholds vary with the matrix vegetation and its utility for the relevant pollinator species. Similarly, for the mitigation services modeled here, there is not a consistent relationship between patch size and effectiveness of service provision. While patch size is an important control, the relationship is complicated by interaction of factors present in a real landscape, such as soil type, forest type, age, and planting density, subsurface drains, slope position and angle, as well as area routed and management on that land. Even within a given site, variation in soil hydraulic behavior with seasonal and diurnal changes and antecedent conditions can be considerable, and mitigation effectiveness will also be strongly dependent on storm frequency and intensity (Cerdà 1996, Fox et al. 1997, Ziegler et al. 2006, Jackson et al. 2008, Marshall et al. 2009). The size of patch necessary to provide effective mitigation would therefore be best assessed on a case‐by‐case basis. However, although thresholds cannot easily be specified, given that patch width (on the axis of flow direction) is positively associated with hydrological mitigating capacity (i.e., amount of infiltration and pollutant removal), it could be expected that this would degrade with increasing fragmentation. Without accounting for this, we cannot fully replicate the findings of Mitchell et al. (2015b).

### Catchment controls on the relationships between forest area and fragmentation on service provision

Our simulations showed considerable variability in the amount of service provided by each combination of patch size and fragmentation. This scatter is likely to reflect the position of individual patches within the catchment, relative to flow accumulation pathways and other patches providing the same service. The strong influence of patch placement results in the standard deviation of up to ±60% for a given scenario of number and area of forest patches; much greater than ±5% observed for individual scenarios in Mitchell et al. (2015b), who also varied patch size and fragmentation, but did not represent flow pathways. This variability of service provision cannot be modeled without accounting for connectivity, which thus illustrates the importance of using realistic topography in studies examining hydrological services. By superimposing virtual landscape configurations on top of this real topography, we were able to extend the findings of Verhagen et al. (2016) and isolate impacts of landscape configuration from the confounding effects of topography observed in real catchment studies. We also found that the importance of patch placement was increased in smaller catchments with greater topographic complexity. The scatter observed also points to functional redundancy of service provision for a regulating service, since reduction of forest area or patch number may not reduce service provision, depending on the landscape configuration and effectiveness of individual patches. The insights explored here enable generalizations that may be important in guiding policy and landscape planning at larger scales.

Location within the landscape is critical for flow‐based services (Eigenbrod et al. 2010, Villa et al. 2014). Based on common assumptions, a complete riparian buffer would provide most mitigation, and riparian buffering is often identified as an effective means of mitigation for diffuse pollutants or sediments (e.g., Mayer et al. 2007). However, by applying random placement, our study showed that mean forest distance to stream per se was not a significant predictor of the amount of service provision until edge density was accounted for. Topographic variation means that placing a forest patch close to a stream does not automatically place it in the site with greatest hydrologically contributing area, since this varies significantly between individual riparian zones. Given that continuous riparian planting is not always possible, and that the service potential of a narrow riparian buffer can be overwhelmed under extreme conditions, these insights highlight the need for explicit hydrological modeling and appropriate interpretation of output to assist spatial targeting for land cover change. Although we only tested two land‐cover types, we would expect our conclusions to hold true over a more complex landscape, since Ziegler et al. (2007) report similar findings across multiple land‐use types with differing levels of mitigation service provision, albeit without representation of flow accumulation.

### Consequences of fragmentation on other services

In this study, we have focused on hydrological mitigating services, however, it is important to consider how fragmentation affects other services. For example, carbon storage and cycling may be strongly affected by microclimate edge effects, which can increase C stocks, making patch size and shape important (Robinson et al. 2009). Fragmentation may deliver benefits from landscape complementation for mobile‐agent‐based ES such as pollination and seed dispersal (Bodin et al. 2006). While potential negative impacts of fragmentation on biodiversity are well documented (e.g., Keller and Largiader 2003, Tabarelli et al. 2008), there may be complementation benefits for species requiring different land cover for feeding and nesting or at different life cycle stages (Fahrig et al. 2011), and poor connectivity also provides barriers to spread of disease. Knowledge on the different patch size thresholds and effects of preferential pathways and landscape complementarity on biodiversity and provision of different ES is therefore of great importance to landscape planning, which must balance the relative importance of these factors for the area under consideration. For catchment level planning, spatially explicit modeling of landscape composition and configuration, such as applied here in the LUCI model, could be used for scoping to identify potential locations for interventions to increase mitigation ES, while minimizing impacts on agricultural production, biodiversity, or other ES.

## Conclusion

Our results show that more complex landscape composition with greater fragmentation can deliver greater ES provision in terms of mitigation of local runoff and accumulated flow of nutrients and sediments. However, any mitigation benefits of fragmentation should be taken into account alongside wider positive and negative implications for other ES, and particularly for biodiversity.

From a landscape planning perspective, these inferences allow us to apply generalized rules that enable large‐scale assessment where explicit spatial modeling is not feasible. This new understanding can also be used to estimate impacts of future scenarios of land cover change (e.g., Fezzi et al. 2015, Harrison et al. 2016). Our finding that the number of patches was more important than distance to stream is particularly important, since it contradicts common assumptions about the universal benefits of riparian planting, and supports a more spatially targeted approach. This new evidence for some benefits of fragmentation on mitigation ES is conceptually useful in directing the way we think about landscape scale land cover change and can help to inform decision making for policy.

## Data Availability

Data are available from the NERC Environmental Information Data Centre (EIDC) at: https://doi.org/10.5285/67f9fe33-14dd-4676-9a6d-65fdbafe2a46
