# Supplementary material for: Fragmentation and thresholds in hydrological flow‐based ecosystem services
Source: Ecol Appl. 2020 Jan 3;30(2):e02046. doi: 10.1002/eap.2046 (PMC7079118; doi:10.1002/eap.2046)

**Supporting Information.** Amy Thomas, Dario Masante, Bethanna Jackson, Bernard Cosby, Bridget Emmett, and Laurence Jones. 2019. Fragmentation and thresholds in hydrological flow-based ecosystem services. *Ecological Applications.*

Appendix S1

*Table S1: metrics of Hiraethlyn sub-catchment*

| Sub-catchment statistics for the Hiraethlyn stage 1 analysis | |
|---|---|
| Area ($km^2$) | 7.6 |
| Number of streams | 36 |
| Drainage density ($km/km^2$) | 1.93 |
| Area draining to each cell in riparian zone ($m^2$) | |
| max | 99,700 |
| min | 25 |
| mean | 2,050 |
| SD | 8,125 |

*Table S2: summary statistics of random and designed landscape compositions tested for the Hiraethlyn.*

| **Number of patches** | **Proportion of catchment** | | | |
|---|---|---|---|---|
| | **0.1** | **0.25** | **0.5** | **0.75** |
| **1** | 5 | 5 | 5 | 5 |
| **2** | 5 | 5 | 5 | 8 |
| **3** | 0 | 0 | 0 | 5 |
| **4** | 5 | 5 | 5 | 6 |
| **5** | 0 | 0 | 1 | 1 |
| **6** | 0 | 0 | 4 | 0 |
| **7** | 0 | 0 | 1 | 0 |
| **8** | 5 | 5 | 3 | 0 |
| **9** | 0 | 0 | 1 | 0 |
| **13** | 0 | 3 | 0 | 0 |
| **14** | 0 | 2 | 0 | 0 |
| **16** | 5 | 0 | 0 | 0 |

*Table S3: correlation between variables tested in regression models for the Hiraethlyn in table 2.*

| column | row | Correlation coefficient | p | Significance |
|---|---|---|---|---|
| Landscape proportion | mean distance to stream | -0.10465 | 3.00E-01 | |
| patch density | mean distance to stream | 0.200085 | 4.59E-02 | * |
| patch density | Landscape proportion | -0.35229 | 3.25E-04 | *** |
| edge density | mean distance to stream | 0.083179 | 4.11E-01 | |
| edge density | Landscape proportion | 0.500506 | 1.14E-07 | *** |
| edge density | patch density | 0.455278 | 1.94E-06 | *** |
| coefficient of variation of Euclidean nearest neighbour distance | mean distance to stream | -0.05071 | 6.16E-01 | |
| coefficient of variation of Euclidean nearest neighbour distance | Landscape proportion | -0.18202 | 6.99E-02 | |
| coefficient of variation of Euclidean nearest neighbour distance | patch density | 0.065587 | 5.17E-01 | |
| coefficient of variation of Euclidean nearest neighbour distance | edge density | -0.20616 | 3.96E-02 | * |

Significance levels: *= 0.05; **= 0.01; ***= 0.001

*Table S4. Regression models relating service provision to landscape composition for all test landscapes (only those which were statistically significant in the Hiraethlyn)*

| **Models of proportion mitigated (variable smoothed by landscape as a factor variable)** | **Direction of coefficient** | **deviance explained %** | **$r^2$** | **AIC** | **significance** |
|---|---|---|---|---|---|
| Landscape proportion | + | 22.1 | 0.217 | 46853.7 | *** |
| patch density | + | 63.1 | 0.629 | 41210.3 | *** |
| edge density | + | 67.4 | 0.672 | 40256.6 | *** |

*Figure S1. Smooth plot for the relationship between the proportion of landscape which is woodland and the area mitigated (the proxy metric to indicate provision of mitigation ES) for simulations across all 10 test landscapes.*

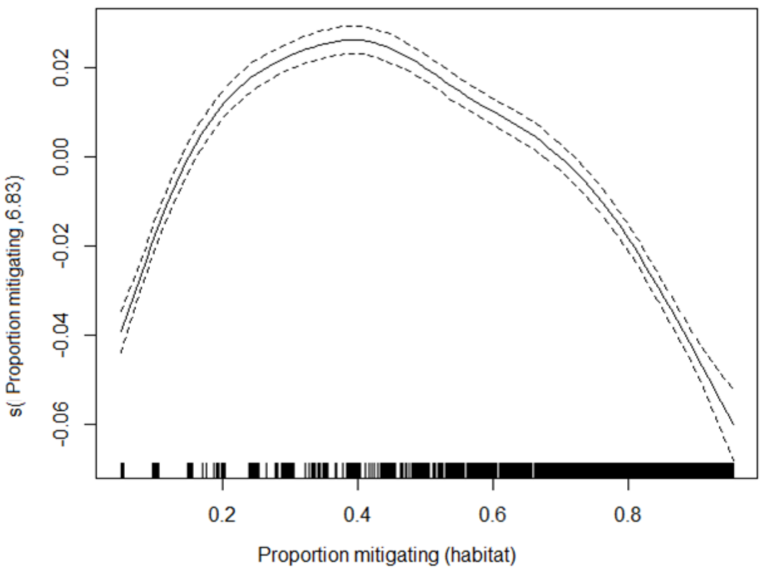

*Figure S2. Plots of the relationship between area mitigated (the proxy metric to indicate provision of mitigation ES) and proportion of catchment as woodland -catchments arranged in order of decreasing size. The inflection point of relationship varies, to suggest that larger catchments had peak service provision at a greater proportion of woodland, and the level of service provision achieved was lower.*

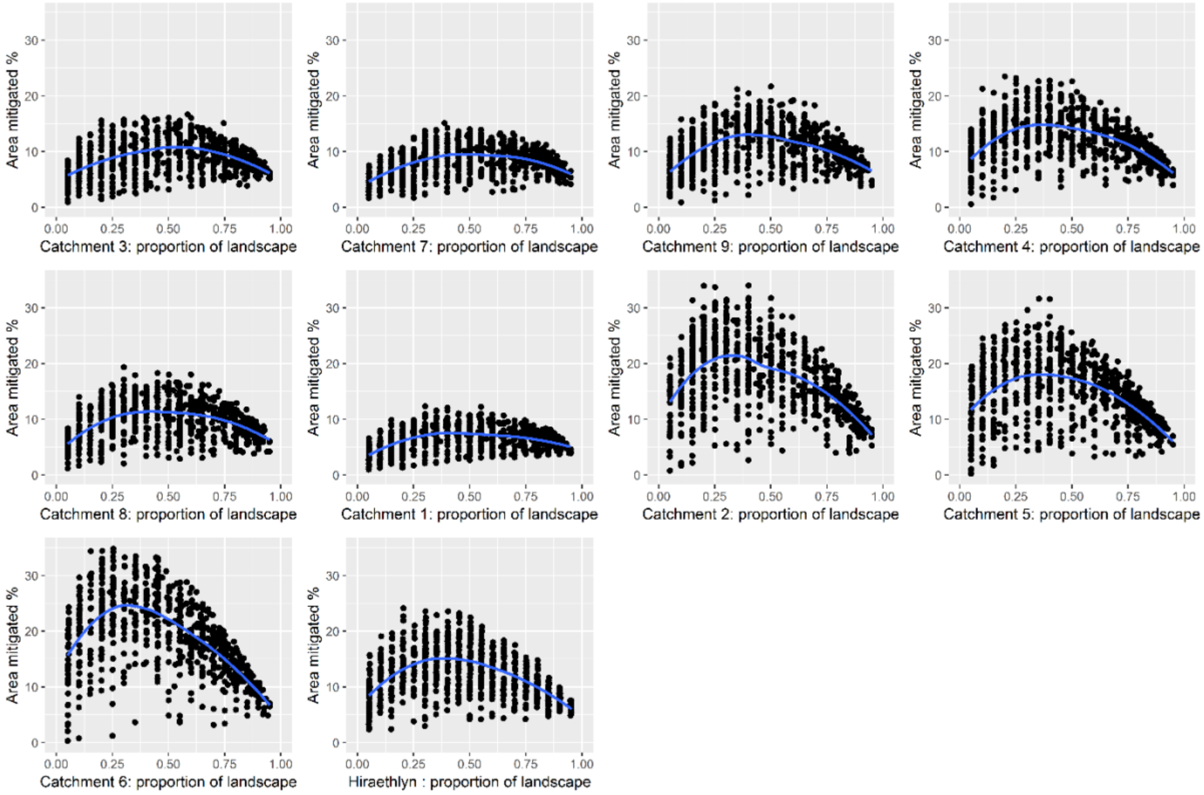

Supplement: Supplementary file 1 [file EAP-30-e02046-s001.pdf]
